# Lives of British Physicians
*The Family Library, No. XIV.


**Published:** 1830-10-01

**Authors:** 


### XVIII.

### LIVES OF BRITISH PHYSICIANS.*

THE lives of British physicians must form a very amusing topic for the youth of these isles. No more will they dream of the gun and the halbert; no more will they think of the valor of Nelson, nor boast of the wooden walls of old England! Napoleon, the invincible, will be forgotten; the star of his destiny, that gleamed so bright and yet so sadly on the Champs de Mars, is a second time set in the shades of the West. Where is Waterloo? Near Brussels. It is pictured no longer in the minds of our nation, and the Duke of Wellington will only be remembered as having been acquainted with Sir Gilbert Blane. Farewel, with Othello, to the pomp and the circumstance of war; farewell to the steed and the clarion; a long farewell to—

The mustering squadron and the clattering car!

The pestle and mortar are in the ascendent; we are henceforth to become a parliament of M. D.'s, and a nation of physicians. Gold headed canes will precede the Chancellor's mace, and the virtues of rhubarb will form the theme of the council-board. Happy, happy, happy doctors, to live in the nineteenth century!

Such were our pleasing reveries on perusing the volume before us, and contemplating the harmless lucubrations of hebdomadal contemporaries. We wish not to disturb their Paradise; we know that where ignorance is bliss 'twere folly to be wise. It is not to be supposed that we intend to give a formal review of the Lives of British Physicians, but an extract or two may prove agreeable to the readers of severer matters. One of the best written lives is that of Dr. Gooch, and the following fragment, which was found amongst some of his loose papers, will furnish an excellent idea of the man.

"From the age of fifteen to twenty-one I was an apprentice to a country surgeon, and when I had nothing else to do, no pills to roll, nor mixtures to compose, I used, by the advice of my master, to go up into my bed-room, and there, with Cheselden before me, learn the anatomy of the bones by the aid of some loose ones, together with a whole articulated skeleton, which hung up in a box at the foot of my bed. It was some time before I overcame the awe with which I used to approach this for-

* The Family Library, No. XIV.

midable personage. At first, even by daylight, I liked to have some one in the room during my interviews with him; and at night, when I laid down in my bed and beheld the painted door which inclosed him, I was often obliged to make an effort to think of something else. One summer night, at my usual hour of retiring to rest, I went up to my bed-room, it was in the attic story, and overlooked the sea, not a quarter of a mile off. It was a bright moonlight night, the air was sultry; and after undressing I stood for some time at my window, looking out on the moonlight sea, and watching a white sail which now and then passed. I shall never have such another bed-room, so high up, so airy, and commanding such a prospect; or, probably, even if I had, it would never again look so beautiful; for then was the spring-time of my life, when the gloss of novelty was fresh on all the objects which surrounded me, and I looked with unmingled hope upon the distant world. Now — but I am rambling from my story. I went to bed, the moonlight which fell bright into my room showed me distinctly the panelled door behind which hung my silent acquaintance; I could not help thinking of him—I tried to think of something else, but in vain. I shut my eyes, and began to forget myself, when, whether I was awake or asleep, or between both, I cannot tell—but suddenly I felt two bony hands grasp my ancles, and pull me down the bed; if it had been real it could not have been more distinct. For some time, how long I cannot tell, I almost fainted with terror, but when I came to myself, I began to observe how I was placed: if what I had felt had been a reality, I must have been pulled halfway out of the bed, but I found myself lying with my head on my pillow, and my body in the same place and attitude as when I shut my eyes to go to sleep. At this moment, this is the only proof which I have that it was not a reality, but a dream."

In the mode of relating this youthful fright, we see the same pensive poetical turn, which threw a charm on the sober writings of the grave physician. We also trace that feeling of disappointment in the midst of success, which formed so prominent a trait in the enthusiastic character of Dr. Gooch, embittering his latter days, and occasioning a *posthumous* effusion of ill-nature, which has thrown almost the only dark shadow on his portrait. To pass to another theme, we recommend the following advice from Dr. Denman to Dr. Parry, to the careful consideration of those who are anxious to get into practice, that is, of every man in the kingdom. Depend upon it that the only way we may succeed in our profession, is really to deserve success. Men may lie, and cringe and flatter themselves into the receipt of gold, but for them the evil day of neglect, and scorn, and the lash of the biographer, is certain to arrive at last. Art and intrigue gain a temporary reputation, perhaps a permanent fortune. But the march of the intriguer is on a bridge as narrow as that of Al Sirat; a single false step may precipitate him into the tortures of ignominy; and even if he prospers in all worldly matters, he carries within him, like Domitian, the terrors of conscious fraud and the presence of an avenging God. Dolum secum, et insidias, et ultorem Deum includit.

"Since the time of your first settling in Bath," writes Dr. Denman to Dr. Parry, "I have ever borne in mind the wish to serve you, if an opportunity offered. There have been very few, but I have mentioned you to several who have come down. I am not surprised that you find your receipts come in slowly at present, but all young practitioners think, when they set up their standard, that the world should immediately flock to it, and they are generally disturbed when they find the contrary. But all business is progressive, and the steps now taken may be so calculated as to produce their effect ten years hence. There must be a vacancy before we can get into business, and when there is, the competition must be equal in many points, as age or standing, character for knowledge, industry or readiness to exert our knowledge for the good of our patients, moral qualities, and the like. On the whole, I do not know what any man can do to get patients, but to qualify himself for business, and then to introduce himself to the notice of those who are likely to employ him. But it is hard to say, on what hinge this matter may turn, as I see men, in great business, of every disposition, or turn of conduct, and with very different degrees of knowledge, and some, I think, with very little, but with great appearance of it. What is very hard, and yet I know two or three instances of it, is, that a man shall be esteemed as a friend, acknowledged to be a man of parts, but none of his friends think of employing him in his profession. This I can hardly explain, unless by the old observation, 'he is too good a poet to be a good physician.' You have judged very wisely in getting appointed to the Charity. It must do some good, though hardly ever so much as is expected from it. I know not why the late Dr. Fothergill said it was a bad thing. With all that can be done, the progress of business must be slow, and may depend upon circumstances which no man can command; but whatever happens, it is a point both of wisdom to the world, and justice to one's self, not to be put out of humour."

